# The Role of Glycemic Variability in Cardiovascular Disorders

**DOI:** 10.3390/ijms22168393

**Published:** 2021-08-04

**Authors:** Valentina Alfieri, Veronika A. Myasoedova, Maria Cristina Vinci, Maurizio Rondinelli, Paola Songia, Ilaria Massaiu, Nicola Cosentino, Donato Moschetta, Vincenza Valerio, Michele Ciccarelli, Giancarlo Marenzi, Stefano Genovese, Paolo Poggio

**Affiliations:** 1 Centro Cardiologico Monzino IRCCS, 20138 Milan, Italy; valentina.alfieri@ccfm.it (V.A.); veronika.myasoedova@ccfm.it (V.A.M.); cristina.vinci@ccfm.it (M.C.V.); maurizio.rondinelli@ccfm.it (M.R.); paola.songia@ccfm.it (P.S.); ilaria.massaiu@cardiologicomonzino.it (I.M.); nicola.cosentino@ccfm.it (N.C.); donato.moschetta@ccfm.it (D.M.); vincenza.valerio@ccfm.it (V.V.); giancarlo.marenzi@ccfm.it (G.M.); stefano.genovese@ccfm.it (S.G.); 2 Dipartimento di Scienze Farmacologiche e Biomolecolari, Università degli Studi di Milano, 20122 Milano, Italy; 3 Dipartimento di Medicina Clinica e Chirurgia, Università degli Studi di Napoli Federico II, 80138 Napoli, Italy; 4 Chirurgia ed Odontoiatria, Dipartimento di Medicina, Università degli Studi di Salerno, 84084 Salerno, Italy; mciccarelli@unisa.it

**Keywords:** glycemic variability, diabetes, cardiovascular disease, translational studies

## Abstract

Diabetes mellitus (DM) is one of the most common and costly disorders that affect humans around the world. Recently, clinicians and scientists have focused their studies on the effects of glycemic variability (GV), which is especially associated with cardiovascular diseases. In healthy subjects, glycemia is a very stable parameter, while in poorly controlled DM patients, it oscillates greatly throughout the day and between days. Clinically, GV could be measured by different parameters, but there are no guidelines on standardized assessment. Nonetheless, DM patients with high GV experience worse cardiovascular disease outcomes. In vitro and in vivo studies showed that high GV causes several detrimental effects, such as increased oxidative stress, inflammation, and apoptosis linked to endothelial dysfunction. However, the evidence that treating GV is beneficial is still scanty. Clinical trials aiming to improve the diagnostic and prognostic accuracy of GV measurements correlated with cardiovascular outcomes are needed. The present review aims to evaluate the clinical link between high GV and cardiovascular diseases, taking into account the underlined biological mechanisms. A clear view of this challenge may be useful to standardize the clinical evaluation and to better identify treatments and strategies to counteract this DM aspect.

## 1. Introduction

Diabetes mellitus (DM) is a well-known risk factor for CVD [1,2,3]. Based on the Framingham risk score, which is one of the most commonly used algorithms to estimate the 10-year cardiovascular risk of an individual, DM patients show an increased incidence of cardiovascular events and have a 2 to 4-fold higher risk of cardiovascular mortality [4,5]. Recently, attention has turned to glucose variability as an independent risk factor underlying CVD risk in addition to hyperglycemia per se.

There are three main components of dysglycemia in DM patients: chronic hyperglycemia, hypoglycemia, and glycemic variability (GV). The clinical term GV biologically refers to the blood glucose oscillations that occur throughout the day (short-term GV), including hypoglycemic periods and postprandial glucose increases, as well as the blood glucose oscillations that occur at the same time on different days (long-term GV). Both short- and long-term GV have been hypothesized to be deleterious [5,6]. Particularly, several studies demonstrated that prolonged poor glycemic control is associated with worse outcomes in CVD patients [7]. Recent studies showed that glucose oscillations were more significantly associated with atherosclerotic-related diseases than chronic hyperglycemia in patients with type 2 DM (T2DM) [8,9,10]. Moreover, high GV, in patients with acute coronary syndrome (ACS), was associated with increased major adverse cardiovascular (MACE) and cerebrovascular events (MACCE) [11,12]. High GV, experienced by patients after cardiac surgery, led to major complications and adverse outcomes [13,14]. The mechanisms that might explain the deleterious effects of GV on cardiovascular complications could be related to oxidative stress, with increased production of reactive oxygen species (ROS), coagulation, vascular inflammation, and endothelial dysfunction [4,6,15]. Indeed, glycemic oscillations were shown to cause significant oxidative stress and inflammation in endothelial cells, to increase the adhesion of monocytes to endothelial cells, and to increase endothelial cell apoptosis [15]. Consequently, current treatment recommendations for patients with DM place a heavy emphasis on closely monitoring and controlling glycemic levels to improve CV outcomes. Intriguingly, new continuous glucose measuring (CGM) technologies are enabling clinicians to collect unbiased glucose data under routine living conditions [16]. CGM data have revealed that (i) glycemia is a strictly stable parameter in healthy subjects, and (ii) persistent aberrant glucose fluctuations are well monitored by these new applicable devices [16].

In light of the above, the present review aims to clarify our knowledge about the role of GV in cardiovascular disease onset and progression. Furthermore, we focused on the importance of identifying and adopting correct therapeutical strategies to obtain GV effective reduction and thus, a beneficial translation on CV outcomes.

## 2. Glycemic Variability and Clinical Implications

Despite several epidemiological investigations that identified chronic hyperglycemia as a risk factor for DM complications leading to CVD, GV has been presented as an independent risk factor and predictor of worse cardiovascular outcomes [17,18]. It is well established that hyperglycemia accelerated glycation, causing a strong relationship between hemoglobinA1c (HbA1c) levels and glucose plasma levels; instead, glucose oscillations are partially independent of blood glucose level and HbA1c. Thus, the “gold” standard HbA1c used to reflect the severity of hyperglycemia could only represent the long-term blood glucose control but not the oscillations in plasma glucose [19]. Studies have shown that the risk of development or progression of macrovascular and microvascular complications are different even though the patients have the same HbA1c level [3,20,21]. According to these studies, mean blood glucose and pre-prandial and post-prandial blood glucose, but not HbA1c, were significantly correlated with the risk of cardiovascular complications [3,20,21]. Accordingly, post-prandial hyperglycemia has been identified as an independent risk factor for CVD complications in DM patients [22], sustaining the relationship between GV and worst DM complications. 

Nowadays, there are many different indexes applied for GV measurement (Table 1), and the standard deviation (SD) of the mean glucose value is one of the most utilized [20]. Other commonly used indicators include the coefficient of variation (CV, calculated as SD/mean glucose value), the mean amplitude glycemic excursion (MAGE), the glycemic lability index (LI), the mean of daily difference (MODD), the continuous overlapping net glycemic action (CONGA), the high blood glucose index (HBGI), the low blood glucose index (LBGI), the interquartile range (IQR), and the Average Daily Risk Range (ADRR) [21]. Recently, it has been recognized that an essential parameter to consider when evaluating GV, which would facilitate safe and effective therapeutic decision making, is also the time spent in the various glycemic ranges (i.e., time per day within target glucose range (TIR), time below target glucose range (TBR), and time above target glucose range (TAR) [23]. Since each index has its limitations, to better compare data across different studies, the clinical community should design standardized guidelines to precisely measure GV [24]. Achieving this goal will be a key point to identify the biological mechanisms underlying GV.

Nevertheless, GV has been evaluated in numerous clinical trials and associated with the negative outcomes in CVD (Table 2). In particular, it was presented as a potential and independent prognostic predictor in patients with chronic and acute coronary artery dis-ease (CAD) [12,25,26], playing an important role in the pathogenesis of atherosclerosis [25,27,28].

### 2.1. Role of Glycemic Variability in Subclinical Atherosclerosis and CVD Risk

First of all, it has been shown that GV has a detrimental effect on subclinical atherosclerosis before plaque formation. Indeed, a high carotid-intimal medial thickness (IMT), measured in patients without carotid stenosis was independently correlated with MAGE and blood glucose SD [26]. According to other studies, an impaired GV was associated with IMT and left ventricular mass index in short-term DM patients with optimal metabolic control [29]. Of note, the latest meta-analysis, aimed to evaluate the GV effects on CVD risk factors, indicates that GV reduction is accompanied by IMT decline, leading to a decreased risk of acute myocardial infarction (AMI) and stroke [30]. In addition, the authors speculated that GV may affect the phosphoinositide 3-kinase (PI3K)/protein kinase B signal pathway, aggravate glucose tolerance, and increase IMT levels, thus leading to CVD [30]. Recently, a new prospective observational study has been proposed to clarify the relationships between GV, evaluated by continuous glucose monitoring (CGM), the incidence of composite cardiovascular events, and the progression of atherosclerosis in patients with type 2 DM who have no apparent history of CVD [31]. Interestingly, the enrollment of 1000 patients is already complete, and hopefully, the results will be available in 2024, after 5 years of follow-up (University Hospital Medical Information Network Clinical Trial Registry UMIN000032325).

### 2.2. Glycemic Variability and Stable Coronary Artery Disease

In stable CAD patients, pre-procedural GV, assessed by CGM, was found to correlate with myocardial and renal damage markers, e.g., increased levels of neutrophil gelatinase-associated lipocalin and serum creatinine, after coronary stenting [41]. GV measured by MAGE before percutaneous coronary intervention (PCI) was associated with increased coronary neointimal growth after 9 months of follow-up [42]. The authors suggested that measuring GV might be useful in secondary prevention, since GV affects neointimal thickening independently of dyslipidemia control [42]. Furthermore, intraoperative GV may negatively influence the outcomes after cardiac surgery. Indeed, recent evidence recognized GV as an independent risk factor for early postoperative acute kidney injury [43]. In addition, for patients who underwent scheduled coronary artery bypass grafting (CABG) surgery, post-operative GV was associated with poor short-term outcomes, including increased risk of post-operative atrial fibrillation (AF), cardiac arrest, pneumonia, renal failure, stroke, sepsis, reoperation, and mortality [13,44,45,46]. Similar, to CABG surgery, in patients who underwent transcatheter aortic valve implantation (TAVI), post-procedural GV was independently correlated with an increased risk of major complications within 30 days after the procedure [14].

### 2.3. Glycemic Variability and Coronary Plaque Vulnerability

It is important to consider that blood GV is also related to the vulnerability of coronary plaque (Table 3), which in turn is closely associated with the risk of experiencing AMI. Higher blood GV, measured by MAGE in ACS patients, was an independent determinant of increased lipid and decreased fibrous contents with larger plaque burden [44]. Of note, significant correlations of MAGE values with coronary plaque instability were found, and higher GV was more closely linked with the markers of oxidative stress and inflammation as compared to conventional glucose indicators [44]. However, recent data evidenced the relationship of GV with the vulnerability of the coronary plaque, which is seen as an alteration of fibrous and necrotic plaque volume in ACS patients, independently of oxidative stress. Nevertheless, in this study, 8-iso-ProstaglandinF2α was used for oxidative stress evaluation, which may not fully reflect oxidative stress damage in ACS patients [34].

The effect of GV has been also assessed on the morphological features of coronary plaques in CAD patients treated with lipid-lowering therapy. Using MAGE, GV was identified as the only independent predictor of the thin-cap fibro-atheroma, evaluating 166 lesions in 72 CAD patients [47]. Of note, various indexes of GV, such as SD, MAGE, CONGA, and MODD, were all associated with vulnerability of coronary plaque, but MAGE and SD were shown to have the highest correlation in comparison with others [48]. Recently, the impact of CD14++/CD16+ monocytes on plaque vulnerability in DM and non-DM patients with asymptomatic CAD has been evaluated. This study revealed that GV, measured by MAGE, could alter the balance of monocyte subsets, favoring those associated with plaque vulnerability [45].

Nevertheless, further studies are required to find a way to prevent the deleterious effects of GV on plaque formation. In this regard, the design of The Observation of Coronary Atheroma Progression under Continuous Glucose Monitoring Guidance in Patients with T2DM (OPTIMAL) study was recently reported (University Hospital Medical Information Network Clinical Trial Registry UMIN000036721). The results of this important study will reveal if reducing the extent of GV will affect coronary atheroma progression [46].

### 2.4. Glycemic Variability and Acute Coronary Syndromes 

Notably, greater GV, measured by MAGE, in patients with AMI who underwent primary PCI was independently associated with composite MACE (in-hospital and 30 days follow-up) as well as not infarct-related coronary revascularization [12]. Results from other studies suggested that higher GV, in ACS patients, was correlated with a greater incidence of acute kidney injury, AF, longer hospitalization, and MACE during 30 days follow-up [11,49]. ACS patients with higher GV measured by self-measured blood glucose (SMBG) had a 2-fold increased risk of MACE at 6 months follow-up [18]. Therefore, high GV might be an additional parameter to be considered for better risk stratification of patients with ACS. Indeed, recently published results proposed the cut-off of >48.6 mg/dL for GV, as assessed by SD during hospitalization, indicating the strong predictive ability of GV on poor outcomes after 1.5 years follow-up [39]. Of note, the HEART2D study (NCT00191282) failed to demonstrate that post-prandial hyperglycemia is an independent risk factor for cardiovascular disease in DM patients [22]. However, a post-hoc analysis confirmed that targeting post-prandial glycemia, in older T2DM AMI survivors, reduced the risk for a subsequent cardiovascular event [50]. On the other hand, analysis of the effect of tight glycemic control in acute myocardial infarction showed that myocyte progenitor cell number (MPC) and myocyte proliferation significantly increased with the early achievement of tight glycemic control, indicating its beneficial effect on the regenerative potential of ischemic myocardium [51].

Recently, the LIBERATES trial (IRAS ID 223768; Trial Registration: ISRCTN14974233) was designed to investigate the role of CGM to optimize glycemic markers in T2DM patients who experienced AMI. The main hypothesis is that a modern glycemic monitoring strategy would optimize glucose levels in these patients and thus improve their quality of life [52]. Therefore, until the results of this trial are available, the reduction of cardiovascular outcomes achieved by controlling the GV remains to be proved.

### 2.5. Glycemic Variability in Patients with Type 1 Diabetes (T1DM) and Cardiovascular Complications

Recently, Greven et al. [53] showed that mean glucose concentration and HbA1c levels, as well as treatment regimens, were similar between T1DM and T2DM. However, the extent of GV was greater in T1DM rather than T2DM, possibly because of the preserved residual beta-cell function in the latter group [53,54]. 

The association between GV and increased risk of cardiovascular complications, such as cardiovascular autonomic neuropathy (CAN) in T1DM, was exanimated in a recently published systematic review [24]. This review showed high heterogeneity in the methodological approaches of different studies, and thus, it is difficult to compare them and come to an unambiguous conclusion regarding GV implication. Of note, the results from analysis of the associations between CAN, the glycemic control, and cardiovascular risk factors in patients with T1DM and T2DM suggested that CAN is a more frequent complication in T1DM [55]. Whereas the attempt to assess whether GV can independently contribute to the CAN onset or progression in T1DM patients could not lead to an unequivocal conclusion due to high heterogeneity and inconsistency of methodological approaches for the CAN and GV assessment in different studies. Thus, further studies using unified and standardized methods to measure CAN and GV are required.

### 2.6. Possible Pharmacological Treatment to Control High Glycemic Variability Detrimental Effects

New glucose-lowering drugs, with a potential benefit on the stabilization of glycemic fluctuations, have been recently introduced for the management of diabetic patients, namely, Glucagon-like peptide 1 receptor (GLP1-R) agonists, sodium-glucose co-transporter 2 (SGLT2) inhibitors, and dipeptidyl-peptidase-4 enzyme (DPP-4) inhibitors.

Nusca et al. [54] summarized the clinical studies investigating the effects of these new agents on GV assessed by CGM. Of note, GLP1-R agonists, SGLT2 inhibitors, and DPP-4 inhibitors seem to be able to alleviate GV with a low risk of hypoglycemia [6,54]. Several studies indicated an important effect of DPP-4 inhibitor on GV reduction, while only a neutral effect was observed on cardiovascular outcomes. On the other hand, SGLT2 inhibitors and GLP1-R agonist was shown to be effective not only on the attenuation of GV but also on the reduction of major adverse cardiovascular events (MACE) in patients with type 2 diabetes [54]. In particular, Famulla et al. [49] showed that Empagliflozin, a potent and selective SGLT2 inhibitor, decreased glucose exposure and variability, increasing time in the glucose target range. Moreover, treatment with Dapagliflozin, over 24 weeks, improved time in range, mean glucose, and glycemic variability without increasing the time spent in the hypoglycemic range [56]. Considering the complex beneficial effect of SGLT2 inhibitors and GLP1-R agonists on both GV and CV outcomes further supports that this pharmacological strategy is more appropriate for the T2DM patient treatment. 

Furthermore, glargine, and the new long-acting insulin analogs degludec, have been proven to be effective strategies for reducing GV [15,57,58]. Nevertheless, no significant difference was found between glargine and degludec in impact on GV [59] and MACE [60]. The main method to control GV is represented by a proper CGM strategy, which combines patient education with the use of effective GV-reducing drugs [49,56,61,62]. Taking these data together, we can conclude that CGM combined with new glucose-lowering drugs has beneficial effects on metabolic control in both T1DM and T2DM as well as across various insulin treatment regimens [49,56,61,62].

Finally, multiple modifiable risk factors should be considered in the evaluation of diabetes complications. Indeed, the result of a recently published clinical trial (NCT00535925), conducted on high-risk diabetic patients with cardiovascular disease, showed that a comprehensive and multifactorial intensive treatment of main cardiovascular risk factors significantly reduces the risk of MACEs and all-cause mortality [63]. 

## 3. Animal Models of Glycemic Variability

**Figure 1:**
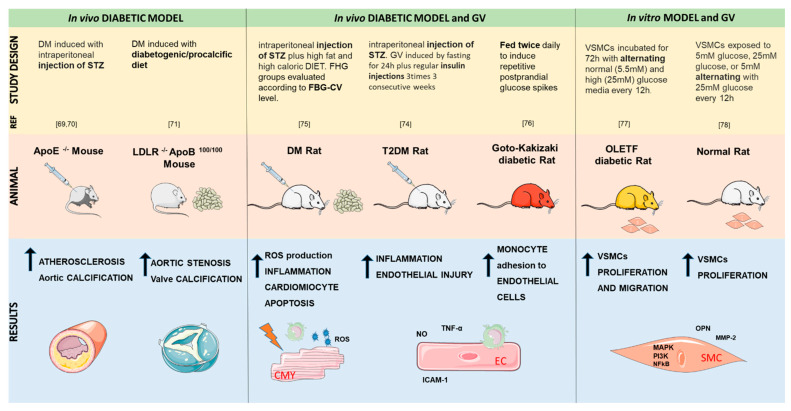
In vivo mouse and rat models used to study the effects of glucose in the cardiovascular system and the respective obtained results.

There are many animal models to study the DM and its complications with characteristics similar to humans; thus, the genetic background and the experimental procedure, as well as the nutritional regimen, should be carefully selected depending on what aspects of the disease are being studied (Figure 1) [57,64,65].

In vivo studies have shown that the earliest defects that characterize DM are abnormalities in myocardial substrate metabolism (increased fatty acid oxidation and decreased glucose uptake and oxidation) and energy metabolism (decreased mitochondrial function) [58,66,67]. Moreover, impaired insulin signaling in the myocardium increases the susceptibility to ischemia and hypertrophy [67,68]. Likewise, atherosclerotic apolipoprotein-E-deficient mice streptozotocin (STZ) injected to induce DM exhibited increased atherosclerosis in the aortic sinus, carotid artery, and abdominal aorta, as well as calcifications in the proximal aorta [69,70]. Finally, the LDLr-/-ApolipoproteinB100/100 mouse model, on a diabetogenic/pro-calcific diet, had a 77% incidence of hemodynamically significant aortic stenosis and developed calcification in both valve leaflets and hinge regions [71].

On the other hand, the animal models used to investigate the effects of GV are generated using artificial interventions, such as poorly maintained insulin control, feeding maltose, or glucose injection [72,73,74]. Indeed, Saito et al. [75] showed that the glucose fluctuations induced in diabetic rats increased ROS production with a direct consequence on the upregulation of thioredoxin-interacting protein—a ubiquitously expressed protein that binds and inhibits thioredoxin thereby inducing oxidative stress and apoptosis, inflammation, and cardiomyocyte apoptosis. Based on these results, the authors speculated that these mechanisms could be at the core of cardiac fibrosis caused by GV. In another study, it has been shown that GV promoted the development of endothelial dysfunction, in a T2DM rat model, via inflammation [74]. In particular, in the serum of DM rats, tumor necrosis factor-alpha (TNF-α) and soluble intercellular adhesion molecule 1 (ICAM-1), inflammatory indicators, were drastically increased in the GV group compared with the steady high glucose group. The results confirmed that high glucose concentrations caused vascular endothelial dysfunction and GV could aggravate the endothelial injury even further. Moreover, in the GV group, there was also an increased production of the vasoconstrictor endothelin-1 (ET-1), indicating that the endothelial vasomotor dysfunction in early DM rats might be related to increasing vessel stress [74]. In agreement, experiments using Kakizaki rats indicated that GV enhances monocyte adhesion to the endothelium in the thoracic aorta [19,76], and in Otsuka Long-Evans Tokushima Fatty (OLEFT) rats, it induces vascular smooth muscle cells proliferation and migration via mitogen-activated protein kinases, PI3K, and nuclear factor kappa B pathways [77]. Furthermore, a subsequent study confirmed that GV enhances vascular smooth muscle cell proliferation to a greater extent than constant hyperglycemia by enhancing matrix metalloprotease-2 (MMP-2) and Osteopontin (OPN) [78].

Taking all this in vivo evidence together, it is clear that DM is strongly associated with the appearance of cardiovascular pathologies and more importantly that GV causes drastic deleterious effects to the vascular system, even compared to the sustained hyperglycemia condition.

## 4. In Vitro Studies of Glycemic Variability Effects on Human Cells

**Figure 2:**
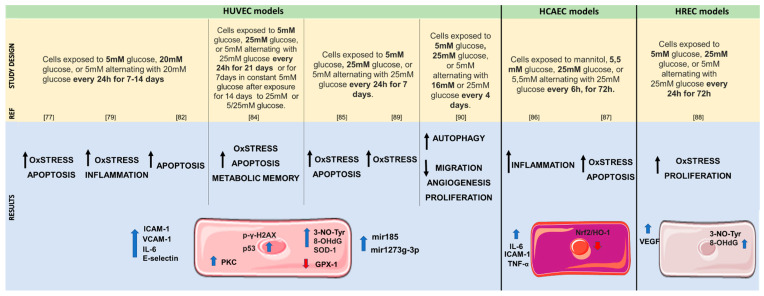
In vitro endothelial models used the effects of glucose in the cardiovascular system and the respective obtained results. HUVEC: human umbilical vein endothelial cells; HCAEC: human coronary artery endothelial cells; HREC: human retinal endothelial cells.

Several lines of evidence suggest that endothelial dysfunction and damage represent the early steps in the development of vascular complications in DM [79,80,81]. Indeed, in vitro investigations on the GV biological effects, in human cells, were primarily focused on endothelial cells (Figure 2). Of note, to reproduce in vitro the GV effects, scientists have adopted glucose oscillation methods that are achieved by continuously changing cell media with different glucose concentrations (i.e., low and high cyclically every 24 h), bearing in mind that in vitro models do not recapitulate all the interday and intraday GV experienced by humans.

Experiments on human umbilical vein endothelial cells showed that glucose oscillations could easily promote nitrotyrosine (3-NO-Tyr) and 8-hydroxydeoxyguanosine(8-OHdG) production by the poly ADP-ribose polymerase pathway [82] as well as promote ROS synthesis by the mitochondrial respiratory chain, enhancing oxidative stress and endothelial cell apoptosis [79]. Simultaneously, the authors revealed an increase in the expression levels of cell-adhesion molecules, such as ICAM-1, vascular adhesion molecule 1 (VCAM-1), and E-selectin, while phosphokinase C (PKC) inhibitor bisindolylmaleimide-I and PKCβ specific inhibitor LY379196 could decrease their expression [19,82,83,84,85]. Experiments on human coronary artery endothelial cells corroborate the above-mentioned findings by showing that glucose oscillations evoked a more intense inflammatory response than constant high glucose, with a marked increase in interleukin-6, TNF-α, and ICAM-1 in supernatants of cell culture [86]. Indeed, coronary artery endothelial cells under oscillating glucose conditions showed an enhancement of oxidative stress and cellular apoptosis through the inhibition of the Nrf2/HO-1 pathway [87]. In human retinal endothelial cells, the exposure to glucose oscillations induced the overproduction of ROS at the mitochondrial transport chain level and an increased production of vascular endothelial growth factor (VEGF) that enhances cell proliferation, leading to the development of diabetic retinopathy [88]. 

Finally, recent evidence indicates that microRNA (miRNA) may function as important regulators in the modulation of DM complications [10]. It has been demonstrated that the exposure of endothelial cells to glucose oscillating conditions produced an impaired antioxidant response increasing the expression of superoxide dismutase-1 (SOD-1), which caused the upregulation of miR-185 that contributes to glutathione peroxidases-1 (Gpx-1) deregulation [89]. In addition, the miRNA-1273g-3p has been linked to increasing autophagy and impaired cell proliferation, migration, and angiogenesis in endothelial cells under oscillating glycemic conditions [90].

These findings suggest that GV may be more closely linked to endothelial dysfunction and cardiovascular events than hyperglycemic conditions. In particular, a deeper analysis of the glucose-oscillation-related molecular mechanisms involved in endothelial damage could be useful for the development of new pharmacological treatments.

## 5. Future Perspectives and Conclusions

Glycemic variability, measured as glucose oscillations intra- and interday, is often underestimated but remains a relevant aspect in the management of DM patients. It has been extensively shown that both GV and hyperglycemia cause similar detrimental effects on the cardiovascular system. However, it is now recognized that the paths leading to cardiovascular complications of GV and sustained hyperglycemia are quite different. 

Indeed, GV might be also considered as an important parameter to better stratify the cardiovascular risk of these patients. However, the evidence that treating high GV is beneficial in reducing cardiovascular outcomes is still scanty, and the investigations of novel therapeutic approaches for GV control are underway.

This review highlights the importance of designing ad hoc clinical trials with standardize GV measurements, involving CGM systems in cardiovascular patients. Indeed, well-designed studies with advanced monitor devices will reveal the right time and dosage of glucose-lowering agents with beneficial results in terms of GV reduction.

In addition to these clinical aspects, we focused on the essential role of the translational approach to investigate and identify the detrimental molecular mechanisms generated by poor glycemic control. However, most of the in vitro studies were performed on immortalized cells with operator-dependent treatments, not resembling the complex human conditions. Hence, studies on primary cells, co-culture systems, and automated bioreactors will be crucial to improve the knowledge of molecular pathways involved in high GV effects. In turn, these approaches will help to identify new therapeutic strategies aimed to protect cells from high GV harmful effects.

In conclusion, we underline the necessity to (1) standardize and automatize GV measurements to obtain clear and comparable data; (2) integrate clinical, cellular, and molecular aspects linked to high GV on cardiovascular outcomes; and (3) design appropriate studies to refine the therapeutical strategies that effectively reduce the cellular and molecular consequences caused by high GV in DM patients.

## Figures and Tables

**Table 1:** GV index.

GV Index		Definition	Reported Features
ADRR	Average Daily Risk Range	The sum of the daily peak risks for hyperglycemia and hypoglycemia	It is equally sensitive in predicting future episodes of extreme hypoglycemia and hyperglycemia, and it is less sensitive to variability within the target blood glucose range [32]
CONGA	Continuous Overlapping Net Glycemic Action	Intraday (within-day) glycemic variation. The standard deviation of the differences of glucose readings for a defined period of hours	It is a parameter that reflects the variability of blood glucose over a certain time interval [20]
CV	Coefficient of Variation	The extent of variability in relation to the mean of the population. 100 * SD/mean of the observations	Less influenced when comparing data sets with widely different mean glucose values (or HbA1c) [6]
IQR	Interquartile Range	Distribution of glucose data at a given time-point calculated from non-parametric statistics. The difference between the 25–75 percentile.	Plotting the IQR (around the median glucose curve) on a modal day glucose profile makes it is easy to spot what time of day has the most GV and needs attention [33]
LI	Lability Index	It processes three glucose values to calculate a lability value and then moves to the next three glucose values	It can serve as an indicator of patients’ prognosis [34,35]
LBGI/HBGI	Low/High Blood Glucose Index	Implemented by converting glucose values into risk scores. If the risk score is below 0, then the risk is labeled LBGI; if it is above 0, HGBI.	They can assess the risk of severe hypoglycemia or hyperglycemia in diabetic patients [36]
MAG	Mean Absolute Glucose	Absolute differences between sequential readings divided by the time between the first and last blood glucose measurement	This measure includes minor as well as major glucose swings and a time axis as the coordinate; it does not permit assessment of the real magnitude of glycemic excursions but rather their kinetics [37]
MAGE	Mean Amplitude Glycemic Excursion	Average of all blood glucose excursions or swings (peak to trough) that are greater than 1 SD of all measures for a given glucose profile	The most common measure of glucose spikes, swings, or excursions as opposed to glucose dispersion [20]
Mean and SD	Mean and Standard Deviation	The amount of variation or dispersion of a data set. The SD of the data set is the square root of its variance	A variation measure that is the most familiar to clinicians and easy to calculate. Most accurate if values are “normally distributed around the mean,” which is often not the case [33]
MODD	Mean of Daily Difference	Interday (between-day) glycemic variation. The absolute value of the difference between glucose values taken on two consecutive days at the same time	It can be used to assess the continuous changes of blood glucose between different days [20]
TIR	Time in Range	The amount of time that glucose is in the target ranges between 3.9 and 10.0 mmol/L within 24 h	Early studies suggest that time-in-range is just as good a predictor of long-term diabetes complications [23,38]

**Table 2:** Glucose variability and cardiovascular outcomes.

CV Diagnosis	Patients Number	Intervention Type	Glucose Fluctuation Monitoring	Observed Effect(s)
ACS STEMI	237	p-PCI	MAGE, SMBG within 72 h after p-PCI	Increased GV associated with increased composite MACE and non-IRA revascularization during in-hospital and 30-day follow-up [12]
ACS STEMI, NSTEMI, UA	864	PCI/CABG	Mean and SD of blood glucose during hospitalization	Increased GV associated with 30-day increased incidence of MACCE and AF during hospitalization, and length of hospital stay [11]
ACS + DM	262	PCI/CABG	Mean and SD of blood glucose 6-months follow-up using the WeChat application	Increased GV associated with 2-fold increased MACE after 6 months of follow-up [18]
ACS + DM STEMI, NSTEMI	327	PCI/CABG/medical treatment	SD with the cut-off > 2.7 mmol/L, in hospital	A GV cut-off value of >2.70 mmol/L predicts mid-term MACE in patients after 16.9 months of follow-up [39]
CAD	1461	CAGB	Post-operative CV within 24 h	Increased post-operative GV associated with increased risk for in-hospital major adverse events [13]
CAD	2073	CAGB	Post-operative SD, CV, MAGE within 24 h	Increased 24 h post-operative GV was independently associated with AF incidence [40]
CAD + DM	28	PCI	SD, CV, MAGE, CONGA 12 h before and after PCI	Altered GV indexes associated with post-procedural impairment of renal function and myocardial damage [41]
CAD + DM	50	PCI	MAGE, 3 consecutive days before PCI	Larger glucose fluctuation is an independent risk factor for impaired uniform vessel healing after second-generation drug-eluting stent implantation after 9 months of follow-up and associated with MACE [42]

ACS: acute coronary syndrome; AF: atrial fibrillation; CABG: cardiac artery bypass grafting; CAD: coronary artery disease; CONGA: continuous overall net glycemic action; CV: cardiovascular; DM: diabetes mellitus; GV: glycemic variability; IRA: infarct-related coronary artery; MACE: major adverse cardiovascular events; MACCE: major adverse cardiovascular and cerebrovascular events; MAGE: mean amplitude of glycemic excursions; NSTEMI: non-ST segment elevation myocardial infarction; p-PCI: primary percutaneous coronary intervention; SD: standard deviation; SMGB: self-measured blood glucose; STEMI: ST-elevation myocardial infarction; UA: unstable angina.

**Table 3:** Glucose fluctuation and plaque vulnerability.

CV Diagnosis	Patients Number	Intervention Type	GV Monitoring	Observed Effect(s)
ACS STEMI, NSTEM	57	PCI	MAGE during hospital admission (at 10 ± 6 days) to minimize the influence of ACS	Higher GV is associated with increased lipid and decreased fibrous contents with larger plaque burden and higher remodeling index [44]
ACS NSTEMI, UA	82	PCI	MAGE, MODD, PPGE, LAGE post-procedural 48–72 h	MAGE and PPGE negatively correlated with the percent fibrous volume and positively with the percent necrotic volume [34]
CAD	72	PCI	MAGE, 3 consecutive days before PCI	Increased GV correlated with lipid-rich plaque formation [47]
CAD	53	PCI	SD, MAGE, CONGA, MODD before the procedure	All GV indexes associated with plaque vulnerability, MAGE, and ST had a higher correlation with coronary plaque vulnerability in comparison to others [48]
CAD + DM	51	PCI	MAGE, 3 consecutive days before PCI	Increased GV correlated with CD14++ CD16+ monocytes in non-DM patientsCD14++ CD16+ monocytes associated with plaque vulnerability [45]

## Abbreviations and Acronyms

3-NO-Tyr	nitrotyrosine
8-OHdG	8-hydroxydeoxyguanosine
ACS	acute coronary syndrome
ADRR	average daily risk range
AF	atrial fibrillation
AMI	acute myocardial infarction
CABG	coronary artery bypass grafting
CAD	coronary artery disease
CGM	continuous glucose monitoring
CONGA	continuous overlapping net glycemic action
CV	coefficient of variation
CVD	cardiovascular diseases
DM	diabetes mellitus
ET-1	endothelin-1
GV	glycemic variability
GPx-1	glutathione peroxidase-1
HbA1c	hemoglobin A1c
HBGI	high blood glucose index
ICAM-1	intercellular adhesion molecule 1
IMT	intimal medial thickness
IQR	interquartile range
LBGI	low blood glucose index
LI	glycemic lability index
MACCE	major adverse cardiovascular and cerebrovascular events
MAGE	mean amplitude glycemic excursion
MMP-2	matrix-metalloprotease-2
MODD	the mean of daily difference
NO	nitric oxide
NSTEMI	non-ST elevation myocardial infarction
OLEFT	Otsuka Long-Evans Tokushima Fatty
OPN	osteopontin
PCI	percutaneous coronary intervention
PI3K	phosphoinositide-3-kinase
PKC	phosphokinase C
ROS	reactive oxygen species
SMBG	self-measured blood glucose
SD	standard deviation of the mean glucose
SOD-1	superoxide dismutase-1
STEMI	ST-elevation myocardial infarction
STZ	streptozotocin
T2DM	type 2 diabetes mellitus
TAVI	transcatheter aortic valve implantation
TNF-α	tumor necrosis factor-alpha
VCAM-1	vascular adhesion molecule-1
VEGF	vascular endothelial growth factor
